# Prospective evaluation of donor-derived cell-free DNA for noninvasive detection of acute rejection in an Asian heart transplant population

**DOI:** 10.3389/ti.2026.16415

**Published:** 2026-10-05

**Authors:** Shanshan Zheng, Sheng Liu, Zhiyuan Zhu, Jie Huang, Zhongkai Liao, Zhe Zheng, Xiaonan Fang, Lin Zhang, Hang Xu, Jiexu Ma, Yige Huyan, Xinhe Xu, Zhengbang Zou

**Affiliations:** Fuwai Hospital, Chinese Academy of Medical Sciences and Peking Union Medical College, Beijing, China

**Keywords:** acute rejection, biomarkers, donor-derived cell-free DNA, heart transplantation, noninvasive monitoring

## Abstract

Endomyocardial biopsy (EMB) is the gold standard for diagnosing post-heart transplant acute rejection (AR) but is invasive. Donor-derived cell-free DNA (dd-cfDNA) is a promising noninvasive biomarker, yet thresholds for Asian recipients remain undefined. This prospective study enrolled 80 patients, with 299 dd-cfDNA specimens analyzed via a novel Chinese-developed next-generation sequencing assay targeting 48 Indel sites. A reference cohort (n = 42) established post-transplant dd-cfDNA kinetics, while a biopsy cohort (n = 41, 18 acute cellular rejection [ACR], 9 antibody-mediated rejection [AMR], 14 no rejection [NR]) paired with EMB evaluated diagnostic performance. dd-cfDNA peaked at week 1, with a stable trend emerging from week 4 and stable levels reached by week 8. AR patients had significantly higher dd-cfDNA levels than NR patients (0.87% vs. 0.23%, P < 0.001). The optimal composite AR threshold was 0.33% (AUC = 0.874, sensitivity 81.0%, specificity 85.7%). dd-cfDNA decreased post-therapy (P = 0.011) and showed an inverse association with left ventricular ejection fraction (LVEF) (P = 0.004). This study provides initial evidence for an assay- and cohort-specific dd-cfDNA threshold (0.33%) for AR detection in Chinese recipients. Given the modest NPV (75.0%), this threshold is better suited for a “rule-in” strategy, and low values should not be used in isolation to defer biopsy. External validation in multicenter cohorts is required before routine clinical implementation.

## Graphical Abstract

**Figure:**
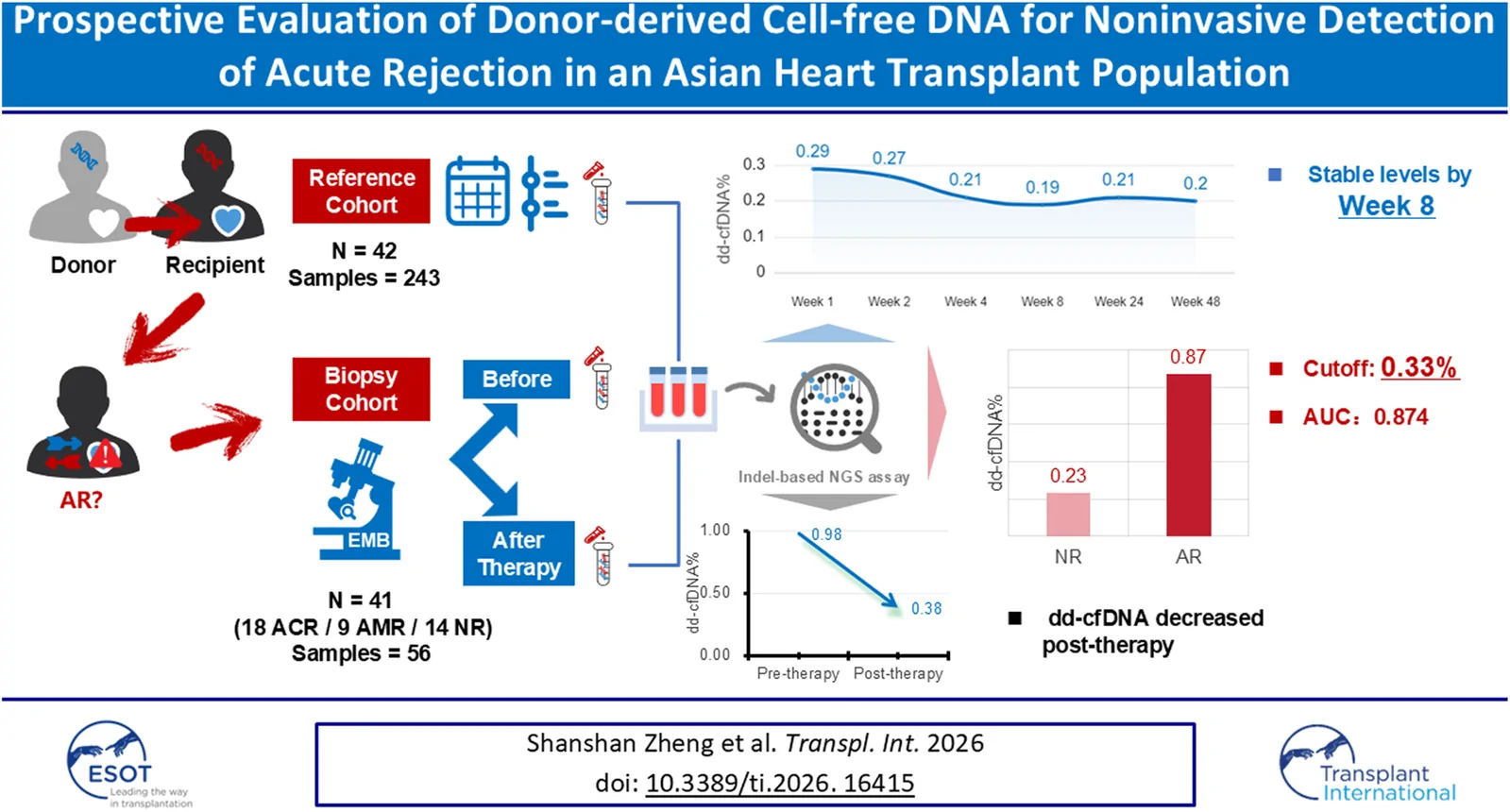

## Introduction

Acute rejection (AR) remains a leading cause of early graft failure after heart transplantation. While endomyocardial biopsy (EMB) is the diagnostic gold standard, its invasiveness and limitations, including sampling error and interobserver variability, are well documented [1–8]. Donor-derived cell-free DNA (dd-cfDNA) has emerged as a promising tool for detecting graft injury [6, 9–12], with international studies validating its diagnostic performance in predominantly Western populations [10, 11]. However, existing diagnostic thresholds are primarily derived from Western populations, and data from Asian cohorts, particularly Chinese heart transplant recipients, are scarce. This gap impedes the clinical implementation of dd-cfDNA in China.

To address this, we conducted the first prospective validation of a domestically developed, Indel-based dd-cfDNA assay in a Chinese cohort [13, 14]. Our study aimed to: (1) define the kinetics of dd-cfDNA post-transplantation; (2) establish assay- and cohort-specific diagnostic thresholds for cellular (ACR) and antibody-mediated rejection (AMR); (3) correlate dd-cfDNA levels with histological severity and graft function; and (4) assess its utility in monitoring therapy.

## Materials and methods

### Study design and population

This prospective, single-center observational study was conducted at Fuwai Hospital, part of the Chinese Academy of Medical Sciences (CAMS). We enrolled two cohorts: a reference cohort (n = 42) of consecutive patients (Oct 2023-Mar 2024) for longitudinal dd-cfDNA monitoring at predefined timepoints up to 1 year (1 week, 2 weeks, 4 weeks, 8 weeks, 24 weeks, and 48 weeks), and a biopsy cohort (n = 41) of patients undergoing EMB for clinical suspicion of rejection. The reference cohort comprised clinically stable patients without signs or symptoms of rejection. All patients in the reference cohort underwent regular post-transplant surveillance, including clinical assessment, laboratory testing, echocardiography, and electrocardiography, with no signs or symptoms suggestive of rejection during the study period. In accordance with our center’s practice, which reserves endomyocardial biopsy for clinical indications due to its invasive nature, protocol (surveillance) biopsies were not routinely performed in this cohort; consequently, the absence of rejection was not systematically confirmed histologically (see Limitations). The clinical indications for for-cause biopsy, which were primarily based on international guidelines, are detailed in Supplementary Table S1 [15]. The study was approved by the Fuwai Hospital Ethics Committee (Approval No. 2023-2131). All organs were voluntarily donated after brain death within China’s national donation system and were allocated through the China Organ Transplant Response System (COTRS). We explicitly confirm that no organs from executed prisoners were used. The research protocol and the organ donation process strictly adhered to the Declaration of Helsinki and the Declaration of Istanbul from both donor and recipient perspectives.

### Histopathological diagnosis

EMB specimens were graded per the International Society for Heart and Lung Transplantation (ISHLT) criteria for ACR and AMR by cardiac pathologists blinded to dd-cfDNA results [16, 17]. Laboratory personnel were likewise blinded to biopsy and clinical data. AR was defined as ACR ≥ Grade 2R and/or AMR ≥ Grade 1. The no rejection (NR) group comprised biopsies with ACR grade 0R and AMR grade 0. For the purpose of rejection subtype analysis, cases of mixed rejection were classified under the AMR group.

### Treatment protocol

Patients with biopsy-proven AR received standardized therapy per ISHLT guidelines and institutional protocols [15, 18]. Treatment was stratified by rejection phenotype, involving pulse methylprednisolone for ACR and combination therapy (e.g., plasmapheresis, Intravenous Immunoglobulin, rituximab) for AMR. Detailed treatment regimens are provided in Supplementary Material, Supplementary Section S2.

### Laboratory methods

Blood was collected in cfDNA BCT tubes (Streck). Plasma was separated by sequential centrifugation, and cfDNA was extracted from 1.8 mL plasma using the QIAamp Circulating Nucleic Acid Kit (Qiagen). dd-cfDNA was quantified using a novel, Chinese-developed NGS assay targeting 48 insertion-deletion (Indel) markers optimized for the East Asian population. The assay demonstrates a linear quantifiable range of 0.05%–1.5% [13, 14], as detailed in Supplementary Material, Supplementary Section S3 and Supplementary Figure S1. Samples with dd-cfDNA levels at or below 0.05% (n = 3) were assigned a value of 0.05% for statistical analysis; no samples were excluded on this basis. Comprehensive methodological details, including primer design, library preparation, sequencing, and bioinformatic analysis, are described in Supplementary Material, Supplementary Section S3.

### Statistical analysis

The primary objective was to evaluate dd-cfDNA’s ability to discriminate acute rejection (AR) from no rejection (NR). Analyses were performed separately for the reference cohort and the biopsy-paired cohort according to their study designs. For the reference cohort (longitudinal repeated measures), temporal dynamics were analyzed using generalized estimating equations (GEE) with an exchangeable correlation structure to account for within-subject correlation and missing data. For cross-sectional group comparisons in the biopsy cohort, each patient contributed only one sample, ensuring independent observations; the Kruskal-Wallis test was used for three or more groups, followed by Dwass-Steel-Critchlow-Fligner post-hoc comparisons, and the Mann-Whitney U test for two groups. Paired pre- vs. post-therapy comparisons were performed using the Wilcoxon signed-rank test. The association between dd-cfDNA and left ventricular ejection fraction (LVEF) was assessed using GEE models, with LVEF treated as both a categorical and a continuous variable, followed by a GEE-based subgroup analysis stratified by rejection status to account for repeated measurements and confounding. Diagnostic performance was evaluated using receiver operating characteristic (ROC) curve analysis, with internal validation via hierarchical (patient-level) bootstrapping (1,000 resamples). No *a priori* sample size calculation was performed; the main diagnostic analysis (21 AR events) and subgroup analyses (particularly for AMR) are based on a limited number of events and therefore should be interpreted as preliminary. These findings provide useful exploratory evidence that merits validation in larger, independent cohorts. All tests were two-sided, with P < 0.05 considered significant. Detailed statistical methods are provided in Supplementary Material, Supplementary Section S4.

## Results

### Study population

From October 2023 to April 2025, 80 heart transplant recipients were enrolled, yielding 299 plasma dd-cfDNA samples. The reference cohort (n = 42) contributed 243 samples, while the biopsy cohort (n = 41) contributed 56 samples paired with EMB.

In addition to the pre-biopsy sample collected for each biopsy procedure, some patients also contributed post-therapy samples (see Figure 1). Detailed patient disposition, including the overlap between cohorts and the distribution of biopsies and samples across groups, is provided in Supplementary Material, Supplementary Section S5.1. Baseline characteristics are summarized in Table 1.

**FIGURE 1:**
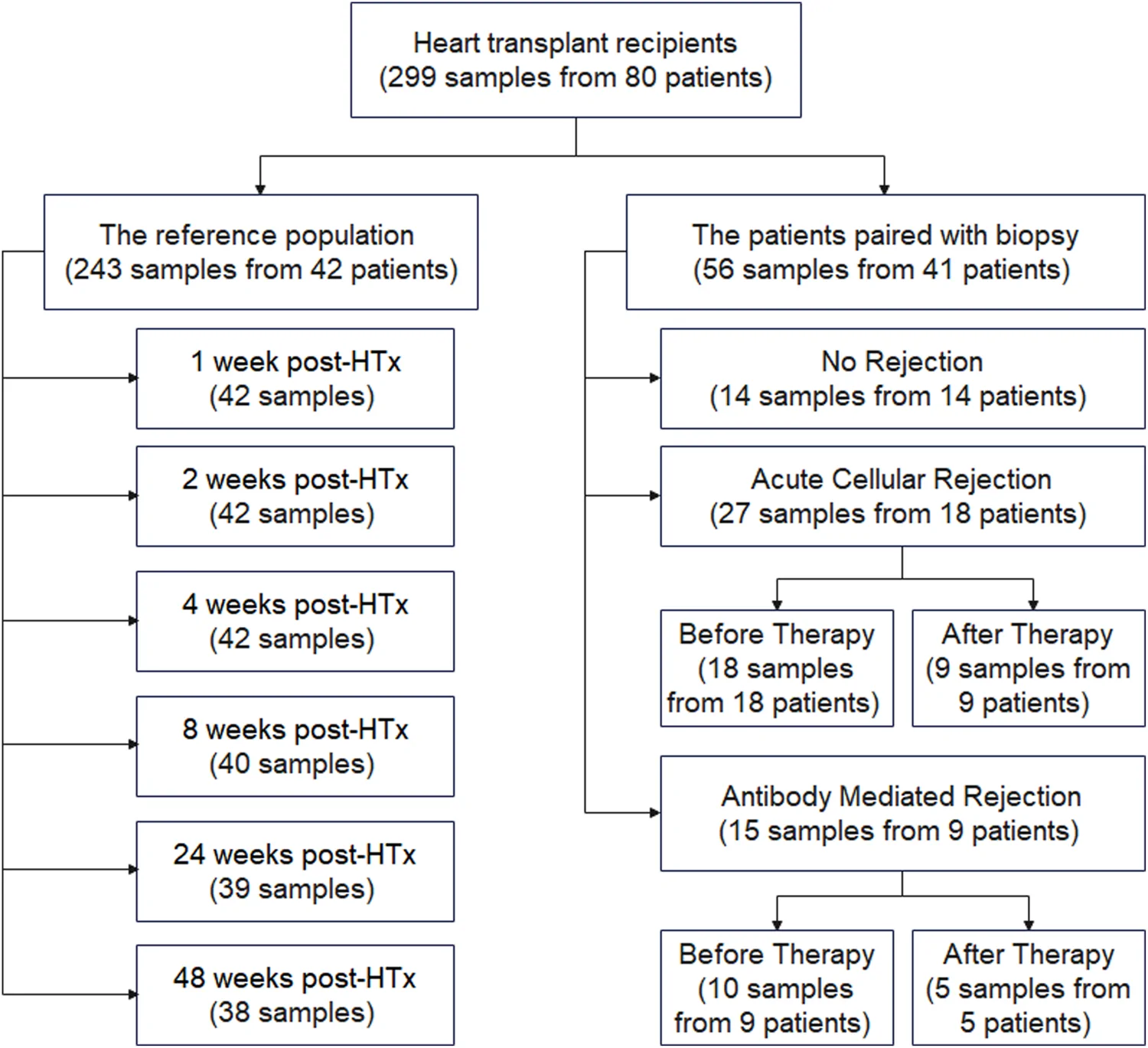
Study enrollment and cohort composition. The flowchart illustrates patient and sample allocation across the reference cohort and the biopsy-paired cohort. In the biopsy-paired cohort, the subset of patients with ACR ≥ 2R or AMR ≥1 received anti-rejection therapy and contributed paired post-therapy samples, with sample numbers as shown in the figure.

**TABLE 1:** Demographic and clinical characteristics of the population.

Variable	Reference cohort	All biopsies cohort	AR (ACR≥2R, AMR≥1)	NR (ACR 0R and AMR 0)	P-value (NR vs. AR)
No. of patients^†^	42	41	20	14	​
Age at enrollment, year	47 (32, 56)	50 (41, 58)	51 (41, 57)	50 (41, 60)	0.944
Days post-transplant at enrollment	7 (7, 7)	722 (15, 1,989)	494 (22, 1,480)	1,063 (15, 2,902)	0.335
Height, cm	169 ± 8	169 ± 8	169 ± 8	169 ± 9	0.879
Weight, kg	61 ± 13	61 ± 12	61 ± 11	60 ± 16	0.831
Female, n%	8 (19.0%)	12 (29.3%)	5 (25.0%)	4 (28.6%)	>0.999
Pretransplant diagnosis, n%		​	​	​	>0.999
Nonischemic cardiomyopathy	35 (83.3%)	34 (83.0%)	16 (80.0%)	12 (85.7%)	​
Ischemic cardiomyopathy	5 (11.9%)	6 (14.6%)	3 (15.0%)	2 (14.3%)	​
Congenital	1 (2.4%)	1 (2.4%)	1 (5.0%)	0 (0.0%)	​
Valvular	0 (0.0%)	0 (0.0%)	0 (0.0%)	0 (0.0%)	​
Other	1 (2.4%)	0 (0.0%)	0 (0.0%)	0 (0.0%)	​
Mechanical support, n%					
None	27 (64.3%)	32 (78.0%)	16 (80.0%)	11 (78.6%)	>0.999
Left ventricular assist device	1 (2.4%)	0 (0.0%)	0 (0.0%)	0 (0.0%)	>0.999
ECMO	1 (2.4%)	0 (0.0%)	0 (0.0%)	0 (0.0%)	>0.999
IABP	14 (33.3%)	9 (22.0%)	4 (20.0%)	3 (21.4%)	>0.999
LVEF at enrollment, %	62 (60, 65)	61 (55, 65)	60 (50, 62)	65 (61, 68)	0.003
PRA positive, n%	5 (11.9%)	8 (18.6%)	4 (20.0%)	2 (14.3%)	>0.999
HLA-I positive*, n%	1 (2.4%)	3 (7.3%)	2 (10.0%)	1 (7.1%)	>0.999
HLA-II positive*, n%	4 (9.5%)	4 (9.8%)	1 (5.0%)	2 (14.3%)	0.555
Donor age, year	43 ± 10	39 ± 10	38 ± 10	39 ± 11	0.648
Donor-recipient blood type incompatibility, n%	7 (16.7%)	3 (7.3%)	2 (10.0%)	1 (7.1%)	>0.999
Sex of donor to recipient, n%		​	​	​	0.293
Male to male	33 (78.6%)	28 (68.3%)	14 (70.0%)	10 (71.4%)	​
Male to female	6 (14.3%)	10 (24.4%)	5 (25.0%)	2 (14.3%)	​
Female to male	1 (2.4%)	1 (2.4%)	1 (5.0%)	0 (0.0%)	​
Female to female	2 (4.8%)	2 (4.9%)	0 (0.0%)	2 (14.3%)	​
Post-transplant mechanical support, n%					
None	33 (78.6%)	31 (75.6%)	14 (70.0%)	12 (85.7%)	0.422
Left ventricular assist device	0 (0.0%)	1 (2.4%)	0 (0.0%)	0 (0.0%)	>0.999
ECMO	0 (0.0%)	3 (7.3%)	3 (15.0%)	0 (0.0%)	0.251
IABP	9 (21.4%)	8 (19.5%)	5 (25.0%)	2 (14.3%)	0.672
Ischemic time, min	198 (166, 355)	268 (192, 359)	311 (208, 358)	209 (172, 350)	0.150
CPB time, min	150 (121, 177)	180 (155, 209)	182 (170, 212)	154 (142, 209)	0.042
Aortic cross-clamping time, min	51 (44, 59)	61 (55, 72)	63 (56, 72)	57 (47, 74)	0.293
Post-transplant CMV antigen positive, n%	2 (4.8%)	4 (9.8%)	2 (10.0%)	0 (0.0%)	0.501
Post-transplant EBV antigen positive, n%	17 (40.5%)	11 (26.8%)	6 (30.0%)	3 (21.4%)	0.704
Post-transplant infection, n%	7 (16.7%)	7 (17.1%)	6 (30.0%)	0 (0.0%)	0.031

^†^ All patient counts represent unique patients.

* HLA-I positive and HLA-II, positive in this table refer to pre-transplant panel-reactive antibody (PRA) positivity, not donor-specific antibodies (DSA).

### Longitudinal dd-cfDNA dynamics

In the reference cohort (n = 42, 243 samples), the overall distribution of dd-cfDNA levels is shown in Figure 2A (median 0.22%, IQR: 0.16%–0.33%). Figure 2B presents boxplots of dd-cfDNA levels at each predefined post-transplant time point. Table 2 summarizes the median values and interquartile ranges: levels peaked at Week 1 (0.29%, IQR: 0.18%–0.40%), gradually declined to a nadir at Week 8 (0.19%, IQR: 0.14%–0.22%), and remained low through Week 48 (0.20%, IQR: 0.10%–0.39%). Figure 2C displays individual patient trajectories over time (gray lines) with the median trend overlaid (red line), illustrating the overall decline and subsequent stabilization.

**FIGURE 2:**
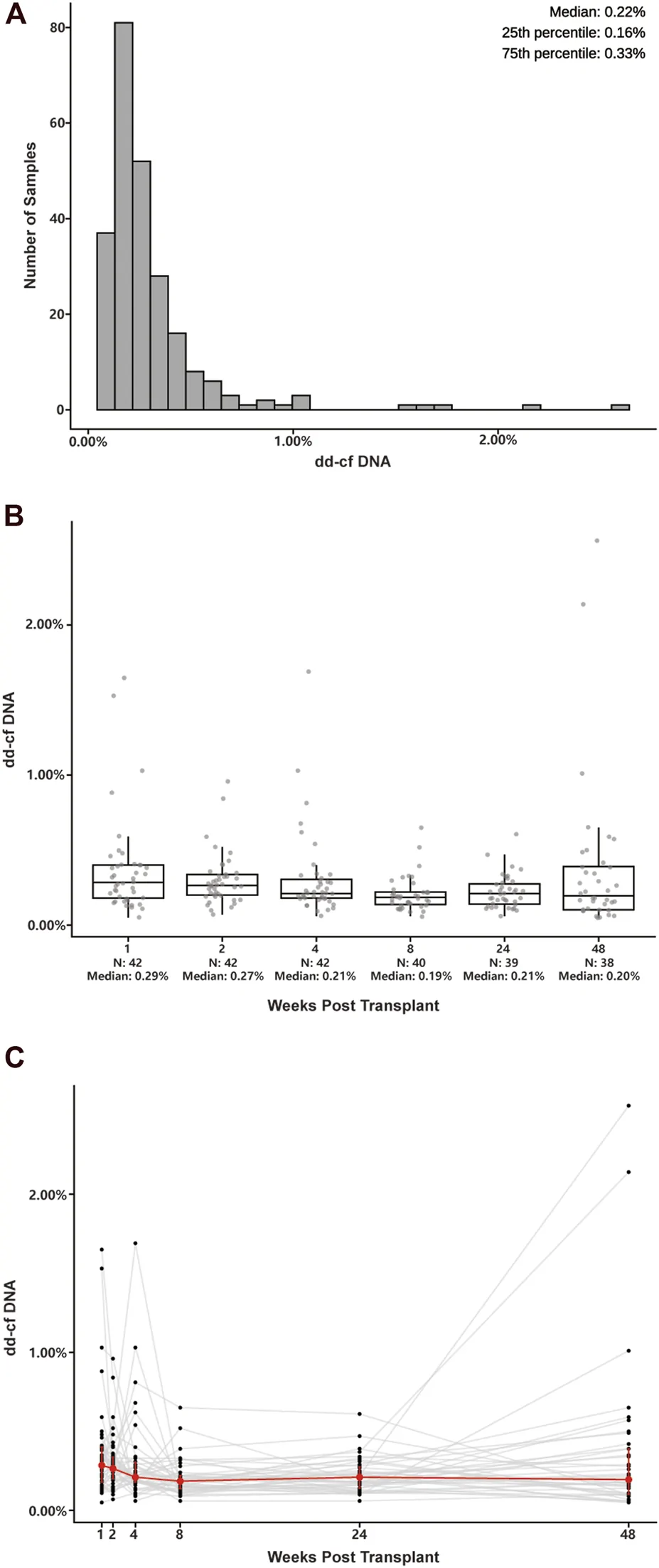
Longitudinal dynamics of dd-cfDNA in the heart transplant reference cohort. **(A)** Histogram showing the distribution of all dd-cfDNA values (n = 243). **(B)** Box plots of dd-cfDNA levels at specific post-operative time points (1, 2, 4, 8, 24, and 48 weeks). **(C)** Line plots of individual dd-cfDNA trajectories over time for all 42 patients.

**TABLE 2:** dd-cfDNA levels across study groups.

Group	Subgroup	No. of samples	Median dd-cf DNA	dd-cf DNA interquartile range (%)	P-value
The reference population	Week 1	42	0.29%	0.18%–0.40%	†
	Week 2	42	0.27%	0.20%–0.34%	​
	Week 4	42	0.21%	0.18%–0.31%	​
	Week 8	40	0.19%	0.14%–0.22%	​
	Week 24	39	0.21%	0.14%–0.28%	​
	Week 48	38	0.20%	0.10%–0.39%	​
The biopsy population	NR (ACR 0R and AMR 0)	14	0.23%	0.18%–0.27%	<0.001
	AR (ACR≥2R,AMR≥1)	21	0.87%	0.35%–1.28%	​
ACR	0R	14	0.23%	0.18%–0.27%	0.014
	1R	7	0.27%	0.16%–0.73%	​
	≥2R	11	0.81%	0.33%–1.03%	​
AMR	0	14	0.23%	0.18%–0.27%	0.008
	1	3	0.87%	0.57%–1.08%	​
	≥2	7	1.04%	0.85%–1.52%	​

^†^ For the reference cohort, pairwise comparisons between time points are described in the Results section (Longitudinal dd-cfDNA, Dynamics); this table presents descriptive statistics only.

We further analyzed the longitudinal dynamics using GEE with an exchangeable correlation structure. The GEE analysis revealed a significant overall time effect (Wald χ^2^ = 15.7, df = 5, P = 0.008). Pairwise comparisons with Holm-Bonferroni adjustment identified significant decreases from Week 1 to Week 8 (mean difference = 0.166%, 95% CI: 0.055%–0.277%, P = 0.042) and from Week 2 to Week 8 (mean difference = 0.095%, 95% CI: 0.033%–0.157%, P = 0.042). No significant difference was observed between Week 4 and Week 8 (mean difference = 0.102%, 95% CI: −0.102%–0.306%, P = 0.330). No significant differences were observed between Week 8 and later time points (Week 24, P = 1.000; Week 48, P = 0.569), nor between Week 24 and Week 48 (P = 0.569), suggesting that week 4 represented the onset of a stable trend, whereas levels had entered a stable range by week 8, although this observation is based on a small cohort and limited follow-up; larger studies are needed to confirm this timing. The estimated marginal means and 95% confidence intervals from GEE are presented in Supplementary Material, Supplementary Section S5.2, Supplementary Table S2.

### Association of dd-cfDNA levels with acute rejection phenotype and severity

A total of 42 endomyocardial biopsies were obtained from 41 patients, including one patient who underwent two biopsies at 2 years and 3 years post-transplant (both confirming AMR grade 2). These biopsies were paired with 56 dd-cfDNA samples for analysis. Based on biopsy findings, the cohort was categorized into four groups: 14 patients/samples with no rejection (ACR 0R, AMR 0); 7 patients/samples with ACR 1R (no AMR); 11 patients with ACR ≥2R (no AMR), paired with 20 samples in total (11 pre-therapy + 9 post-therapy); and 9 patients with AMR (AMR grade 1 or AMR ≥2, including 5 cases of mixed rejection) paired with 15 samples. Among the 21 diagnosed AR episodes, 8 occurred during the first-year post-transplant, 2 during the second year, 8 during years 3–5, and 3 beyond 5 years post-transplant.

The median baseline dd-cfDNA level in the entire biopsy cohort was 0.36% (IQR: 0.23%–0.88%; Figure 3A). Patients with AR exhibited a significantly higher median dd-cfDNA level (0.87%) compared to those without rejection (NR, 0.23%; P < 0.001) (Figure 3B; Table 2). Subgroup analysis by ACR grade showed comparable median dd-cfDNA levels between ACR 1R and ACR 0R biopsies (0.27% vs. 0.23%, P = 0.735). However, dd-cfDNA levels were significantly elevated in biopsies with ACR ≥ 2R compared to ACR 0R (0.81% vs. 0.23%, P = 0.009) (Figure 3C; Table 2). Although numerically higher in ACR ≥ 2R than in ACR 1R (0.81% vs. 0.27%), this difference did not reach statistical significance (P = 0.272; Figure 3C; Table 2).

**FIGURE 3:**
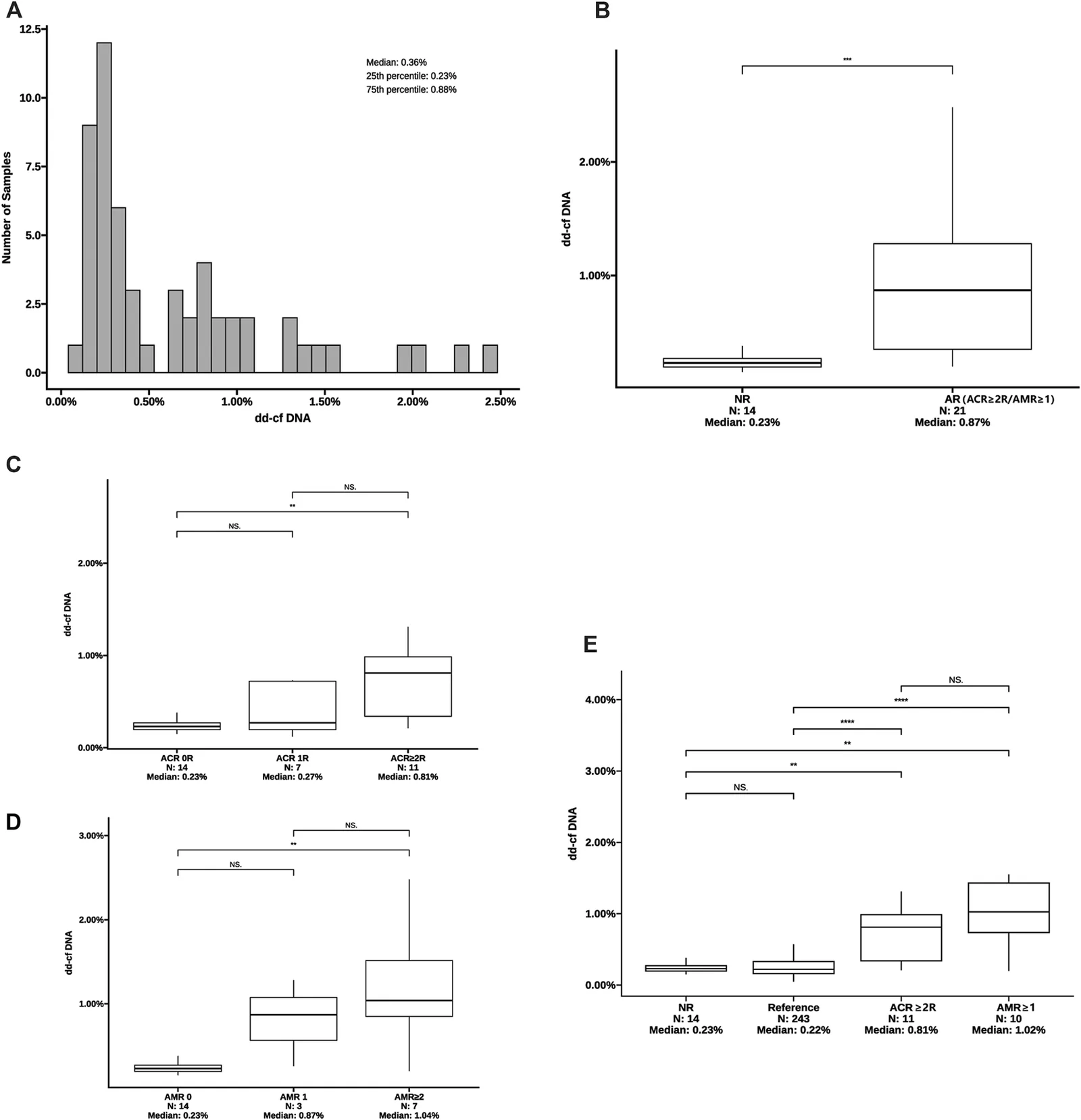
dd-cfDNA levels in the for-cause biopsy cohort: **(A)** overall distribution histogram; **(B)** comparison between acute rejection (AR, defined as ACR≥2R and/or AMR≥1) and non-rejection (NR) groups; **(C)** levels by acute cellular rejection (ACR) severity grade; **(D)** levels by antibody-mediated rejection (AMR) severity grade (including mixed rejection); **(E)** comparison between NR, the reference population, ACR ≥2R, and AMR ≥1 groups. For panels **(B–E)**, each patient contributed one sample; N indicates the number of unique patients. Data are presented as median with sample size indicated.

dd-cfDNA levels also correlated with AMR severity. dd-cfDNA levels in the AMR 1 group were notably higher than those in the AMR 0 group (0.87% vs. 0.23%); however, this elevation did not reach statistical significance (P = 0.159), likely due to limited sample size. Notably, a statistically significant increase was observed in the AMR ≥2 group compared to AMR 0 (1.04% vs. 0.23%, P = 0.013) (Figure 3D; Table 2). No significant difference in dd-cfDNA levels was detected between the AMR ≥2 and AMR 1 groups (1.04% vs. 0.87%, P = 0.705; Figure 3D; Table 2).

At histologically matched severity grades, median dd-cfDNA levels were numerically higher in AMR than in ACR, although these differences did not reach statistical significance, with values of 0.87% in AMR 1 versus 0.27% in ACR 1R (P = 0.948) and 1.04% in AMR ≥2 versus 0.81% in ACR ≥ 2R (P = 0.885). The median dd-cfDNA level in the reference population (0.22%) was nearly identical to that in the NR group (0.23%; P > 0.999; Figure 3E). Notably, both the ACR group (defined as ACR ≥ 2R) and the AMR group (defined as AMR ≥1) demonstrated markedly elevated dd-cfDNA levels compared to both the NR and reference populations (Figure 3E). The wide range of post-transplant time points at biopsy (from <1 year to >5 years) introduces potential confounding due to differential dd-cfDNA kinetics between early and late periods; this is further addressed in the Discussion.

### dd-cfDNA changes after anti-rejection therapy

The dd-cfDNA was measured before and 2 weeks after the anti-rejection therapy. The median dd-cfDNA levels exhibited a significant reduction from 0.98% (IQR: 0.77%–1.35%) at baseline (pre-therapy) to 0.38% (IQR: 0.31%–0.69%) post-therapy (P = 0.011, paired Wilcoxon test; Figure 4A). Meanwhile, among the 14 patients included in this analysis, several key clinical parameters showed significant differences between pre- and post-therapy assessments. NT-proBNP levels decreased substantially from 7884.0 (IQR: 4385.0–15810.5) pg/mL to 3236.0 (IQR: 2113.3–4432.5) pg/mL (P = 0.014; Figure 4C). CD4^+^ lymphocyte counts also declined significantly, from 497.5 (IQR: 254.3–677.3) × 10^6^/L to 275.0 (IQR: 202.8–481.8) × 10^6^/L (P = 0.046; Figure 4D). In contrast, no significant differences were observed in left ventricular ejection fraction (pre-therapy: 60.0% [IQR: 45.5%–61.3%] versus post-therapy: 59.0% [IQR: 52.8%–65.0%], P = 0.565; Figure 4B) or total B cell counts (pre-therapy: 73.5 [IQR: 47.0–156.3] × 10^6^/L versus post-therapy: 60.5 [IQR: 5.5–120.5] × 10^6^/L, P = 0.370; Figure 4E). These treatment-response analyses are based on a small subset (n = 14).

**FIGURE 4:**
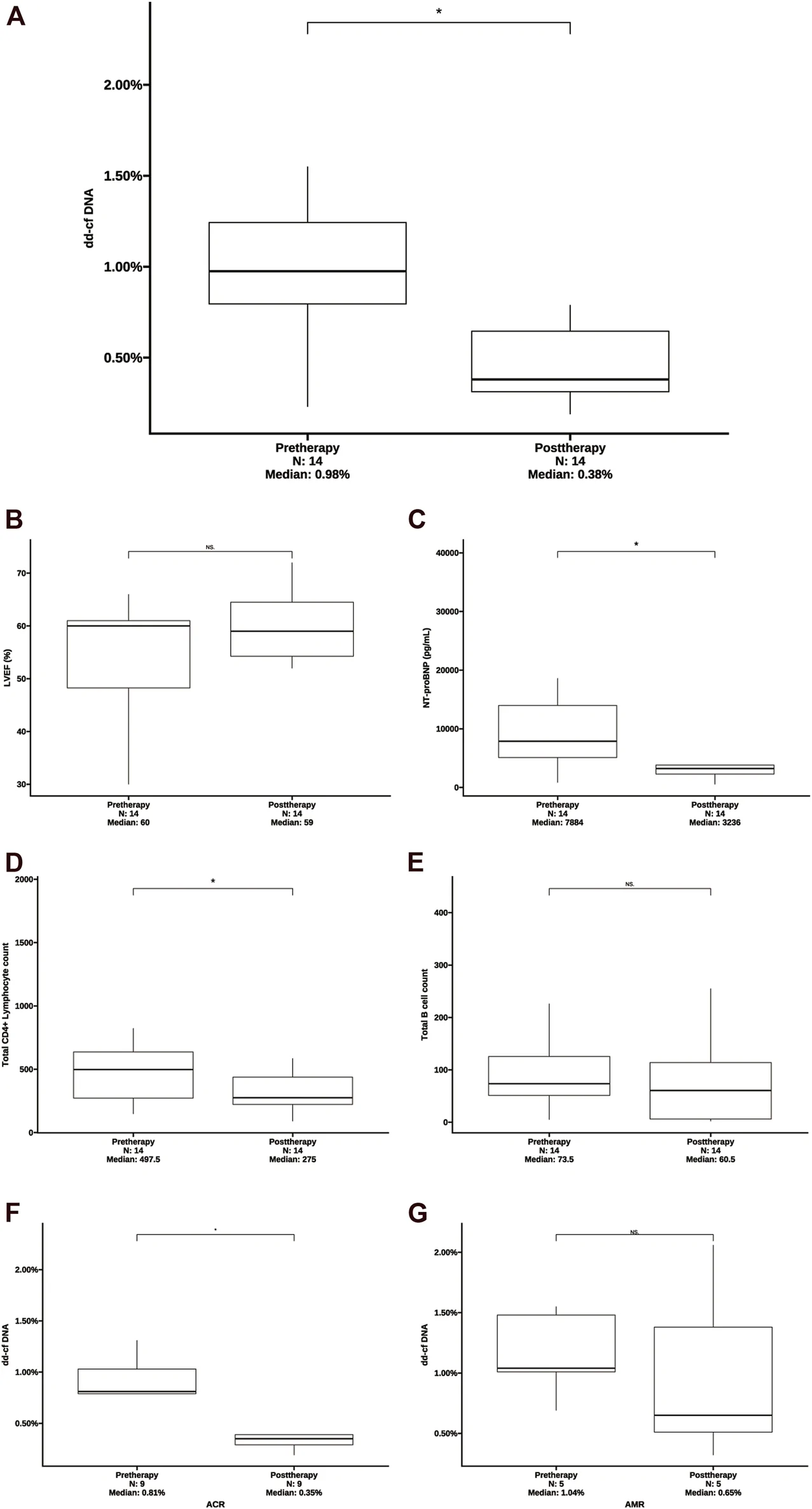
Dynamics of dd-cfDNA and associated clinical parameters in 14 acute rejection patients before and after anti-rejection therapy. Box plots show changes in **(A)** dd-cfDNA, **(B)** left ventricular ejection fraction (LVEF), **(C)** NT-proBNP levels, **(D)** CD4^+^ T-cell counts, **(E)** B-cell counts across all patients, **(F)** dd-cfDNA in patients with acute cellular rejection (ACR), and **(G)** dd-cfDNA in patients with antibody-mediated rejection (AMR). Solid lines represent medians; dashed lines indicate interquartile ranges. *P < 0.05, **P < 0.01 versus pre-therapy.

When stratified by rejection type, a significant reduction in dd-cfDNA levels was observed only in the ACR subgroup. Following anti-rejection therapy, dd-cfDNA levels in the ACR subgroup declined from 0.81% (IQR: 0.57%–1.17%) to 0.35% (IQR: 0.25%–0.51%) (P = 0.011; Figure 4F), whereas in the AMR subgroup, the decrease from 1.04% (IQR: 0.85%–1.52%) to 0.65% (IQR: 0.42%–1.72%) did not reach statistical significance (P = 0.421; Figure 4G).

### Association of dd-cfDNA levels with severity of allograft dysfunction

All dd-cfDNA measurements were collected concurrently with echocardiographic assessments. Stratification of the study cohort by LVEF revealed significant differences in dd-cfDNA levels across groups (P < 0.001, GEE model; Figure 5A). Patients with severely reduced LVEF (<50%; n = 9 measurements from 9 patients) exhibited the highest median dd-cfDNA level (0.79%; IQR: 0.27%–1.03%). Intermediate levels were observed in patients with mildly reduced LVEF (50%–60%; n = 30 measurements from 22 patients), with a median of 0.35% (IQR: 0.25%–0.71%). In contrast, patients with preserved LVEF (≥60%; n = 251 measurements from 68 patients) demonstrated the lowest median dd-cfDNA level (0.22%; IQR: 0.17%–0.34%). In the GEE model with LVEF ≥60% as the reference group, patients with LVEF <50% had significantly higher dd-cfDNA levels (Estimate = 0.00476, SE = 0.002122, Wald = 5.04, P = 0.025). Patients in the 50%–60% group also exhibited significantly higher dd-cfDNA levels compared to the reference group (Estimate = 0.00203, SE = 0.000965, Wald = 4.45, P = 0.035). In GEE models treating LVEF as a continuous variable, a statistically significant negative association was observed between LVEF and dd-cfDNA levels in the overall cohort (Estimate = −0.000143, 95% CI: −0.000240 to −0.000046; Wald = 8.44, P = 0.004; Figure 5B). To evaluate whether this association was confounded by acute rejection, we performed a stratified analysis by rejection status using GEE. In the AR subgroup, the negative association between LVEF and dd-cfDNA remained significant (Estimate = −0.000208, 95% CI: −0.000393 to −0.000023; Wald = 4.90, P = 0.027; Figure 5C), whereas no significant association was detected in the NR subgroup (Estimate = −0.000010, 95% CI: −0.000064 to 0.000044; Wald = 0.14, P = 0.711; Figure 5C). These findings suggest that the overall inverse relationship between LVEF and dd-cfDNA is largely driven by the presence of acute rejection.

**FIGURE 5:**
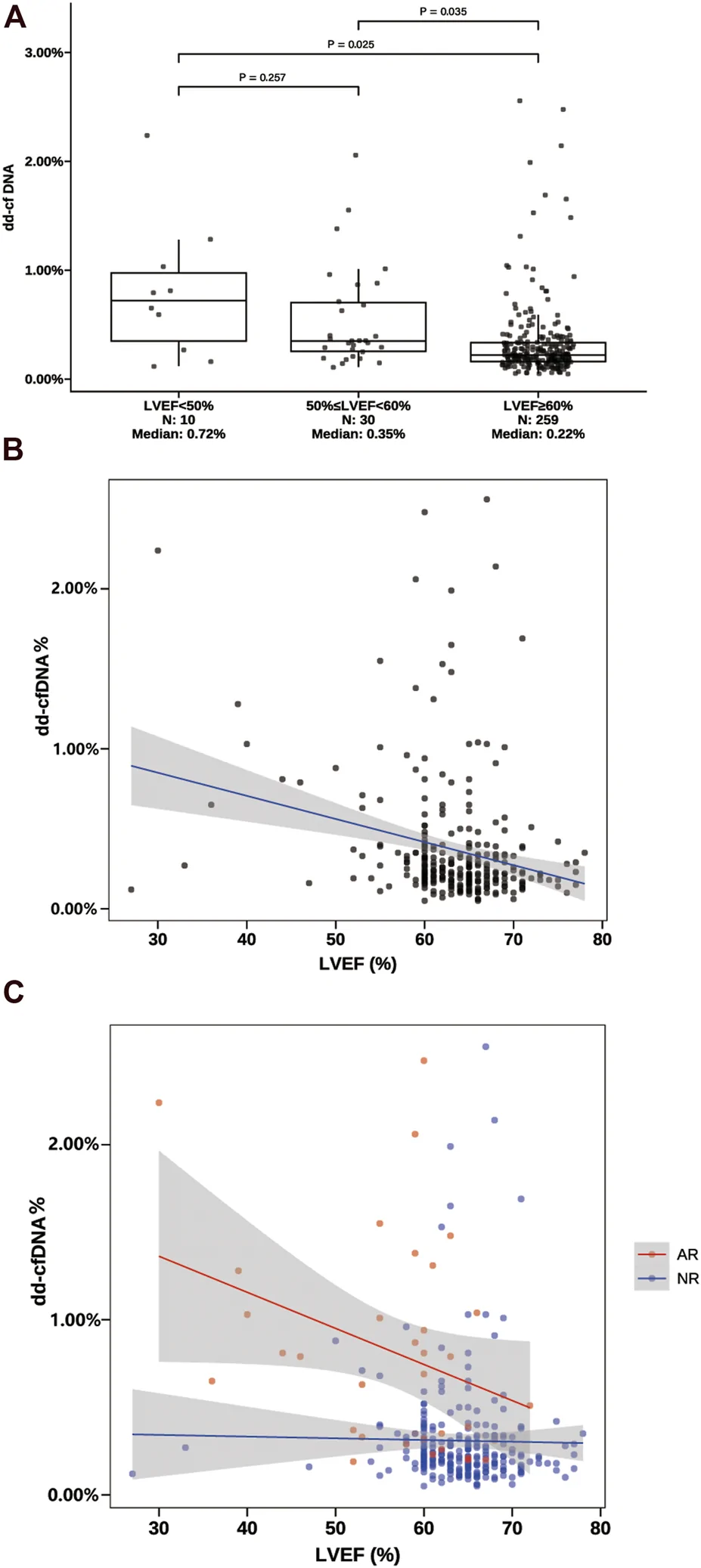
Relationship between dd-cfDNA levels and allograft dysfunction assessed by echocardiography. **(A)** dd-cfDNA levels stratified by left ventricular ejection fraction (LVEF) severity. Boxes represent median and interquartile range; whiskers indicate 1.5×IQR. P value (P < 0.001) was derived from a generalized estimating equation (GEE) model comparing all three LVEF categories. The number of measurements for each group was: LVEF <50% (n = 9 measurements from 9 patients), LVEF 50%–60% (n = 30 measurements from 22 patients), and LVEF ≥60% (n = 251 measurements from 68 patients). **(B)** Association between dd-cfDNA levels and continuous LVEF from GEE regression models. The blue line indicates the linear regression fit. **(C)** GEE-based regression analysis of the association between continuous LVEF and dd-cfDNA levels, stratified by rejection status (AR vs. NR). Error bars represent 95% confidence intervals. AR, acute rejection; NR, no rejection.

To explore discordant results between dd-cfDNA levels and echocardiographic function (as shown in Figure 5A), clinically significant cases are described in detail in Supplementary Table S4. Among 12 patients with elevated dd-cfDNA (0.35%–2.48%) despite preserved LVEF (≥60%), 91.7% (11/12) had biopsy-proven rejection (ACR ≥1R or AMR ≥1), with 66.7% (8/12) exhibiting significant rejection (ACR ≥2R or AMR ≥2). Conversely, among 3 patients with reduced LVEF (27%–47%) but low dd-cfDNA (<0.3%), 66.7% (2/3) had biopsy-confirmed absence of rejection, while one patient had ACR grade 1R. Three patients exhibited elevated dd-cfDNA with preserved LVEF but biopsy findings of no or low-grade rejection with prominent Quilty lesions.

### Performance characteristics of dd-cfDNA to detect AR

In the biopsy cohort, each patient contributed only one dd-cfDNA measurement for the diagnostic performance analysis, which was collected prior to the corresponding endomyocardial biopsy and before any anti-rejection therapy. Thus, the observations were independent, and standard receiver operating characteristic (ROC) curve analysis was performed. ROC curve analysis was used to evaluate the diagnostic performance of dd-cfDNA levels for AR, ACR and AMR, with each phenotype distinguished from the no rejection state (Figure 6A). For AR, the ROC analysis yielded an apparent AUC of 0.874 (95% CI: 0.734–1.000). An optimal dd-cfDNA threshold of 0.33% was identified (Table 3), which achieved a diagnostic sensitivity of 81.0%, specificity of 85.7%, PPV of 89.5% and NPV of 75.0%. For ACR, the ROC curve demonstrated an AUC of 0.832 (95% CI: 0.657–1.000). At its optimal threshold of 0.33% (Table 3), diagnostic sensitivity was 81.8%, specificity was 85.7%, with a corresponding PPV of 81.8% and NPV of 85.7%. For AMR, the ROC analysis showed an AUC of 0.875 (95% CI: 0.715–1.000). Using the optimal threshold of 0.69% (Table 3), diagnostic sensitivity was 80.0%, specificity was 92.9%, yielding a PPV of 88.9% and NPV of 86.7%.

**FIGURE 6:**
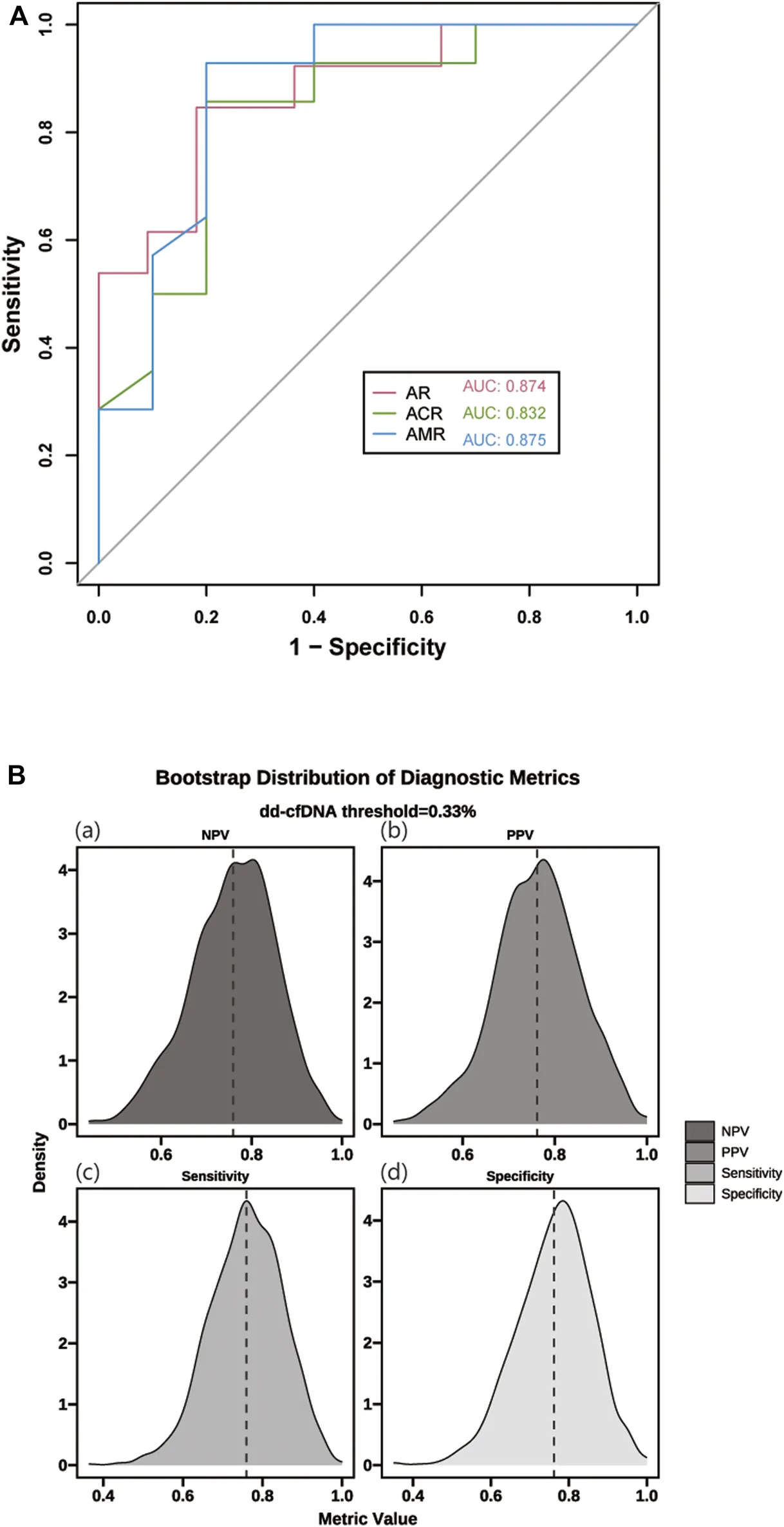
Diagnostic performance of dd-cfDNA for acute rejection detection. **(A)** Receiver operating characteristic (ROC) curves for dd-cfDNA in identifying any acute rejection (AR), antibody-mediated rejection (AMR ≥1), and acute cellular rejection (ACR ≥2R). Area under the ROC curve (AUROC) values are shown. Corresponding ROC characteristics are summarized in Table 3. **(B)** Bootstrap distributions of diagnostic metrics at the 0.33% dd-cfDNA threshold across 1,000 resamples: **(a)** NPV, **(b)** PPV, **(c)** Sensitivity, and **(d)** Specificity. Vertical dashed lines represent 95% confidence interval bounds. Note the bimodal distribution pattern in specificity.

**TABLE 3:** AUCs and 95% CI of dd-cfDNA test and dd-cfDNA optimal cutpoint to detect biopsy-positive acute rejection.

Diagnosis	dd-cfDNA test		Metric score: youden	Cutpoint	TP	FP	TN	FN	Sensitivity	Specificity	PPV	NPV	AUC (95% CI)
	AUC	95% CI											
AR (ACR≥2R,AMR≥1)	0.874	0.734–1.000	0.67	0.33%	17	2	12	4	81.0%	85.7%	89.5%	75.0%	0.833 (0.705–0.962)
			NA	0.69%	15	1	13	6	71.4%	92.9%	93.8%	68.4%	0.821 (0.700–0.943)
ACR (ACR≥2R)	0.832	0.657–1.000	0.68	0.33%	9	2	12	2	81.8%	85.7%	81.8%	85.7%	0.838 (0.685–0.990)
			NA	0.69%	7	1	13	4	63.6%	92.9%	87.5%	76.5%	0.782 (0.618–0.947)
AMR (AMR≥1)	0.875	0.715–1.000	NA	0.33%	8	2	12	2	80.0%	85.7%	80.0%	85.7%	0.829 (0.667–0.990)
			0.73	0.69%	8	1	13	2	80.0%	92.9%	88.9%	86.7%	0.864 (0.716–1.000)

The diagnostic performance of dd-cfDNA for diagnosing AR at the threshold of 0.33% was internally validated using 1,000 hierarchical bootstrap resamples with patient-level block sampling as a sensitivity analysis. This analysis yielded a sensitivity of 75.95% (95% CI: 54.03%–90.91%), specificity of 76.15% (95% CI: 50.00%–90.00%), PPV of 76.20% (95% CI: 52.38%–90.92%), and NPV of 75.91% (95% CI: 52.94%–90.48%). All point estimates clustered within a narrow range of 75.91%–76.20%, while the confidence intervals exhibited substantial width, spanning 36.88% for sensitivity, 40.00% for specificity, 38.54% for PPV, and 37.54% for NPV. The lower confidence bounds reached 54.03% for sensitivity and 50.00% for specificity. Figure 6B presents density plots for sensitivity, specificity, PPV, and NPV with vertical dashed lines demarcating the 95% confidence interval bounds, which revealed near-symmetrical distributions for sensitivity and NPV, a right-skewed distribution for PPV, and a bimodal tendency for specificity with density peaks at 0.6 and 0.8.

## Discussion

This prospective study provides the first evaluation of dd-cfDNA for monitoring acute rejection in a Chinese heart transplant cohort using a domestically developed assay platform. The field of dd-cfDNA research has progressed substantially, with recent investigations exploring diverse methodological approaches and uncovering significant ethnic variations, such as differences between Black and White populations, alongside sex-specific disparities [19–22]. Existing evidence suggests that Black patients experience higher levels of allograft injury and exhibit significantly elevated dd-cfDNA levels post-transplantation compared to their White counterparts [21]. Nonetheless, data concerning dd-cfDNA in the context of heart transplant rejection remain limited among Asian populations, thereby creating a critical knowledge gap within this ethnic group. Consequently, our study was designed to address this unmet need. This prospective study represents the first validation of dd-cfDNA for monitoring acute rejection in a Chinese heart transplant cohort using a domestically developed assay platform.

The diagnostic performance of dd-cfDNA observed in our cohort is broadly consistent with foundational studies such as GRAfT, supporting its potential cross-ethnic applicability as a biomarker for rejection [11]. We determined an assay- and cohort-specific diagnostic threshold of 0.33% for composite acute rejection, which exhibited apparently high diagnostic accuracy (apparent AUC 0.874). This threshold is numerically higher than the 0.20%–0.25% cutoffs established in Western cohorts using a Single Nucleotide Polymorphism (SNP)-based targeted amplification next-generation sequencing (NGS) assay (AlloSure®; CareDx, Inc.). The observed discrepancy may result from several factors: (1) intrinsic ethnic variations in cfDNA metabolism or immunological responses; (2) fundamental methodological differences between our Indel-based NGS assay and the SNP-based AlloSure® platform, particularly concerning background noise and informatics; and (3) potential cohort selection biases. Notably, without an external validation cohort and direct head-to-head comparison with other assays, the relative contributions of biological (ethnic) versus technical (assay-specific) factors to the higher observed threshold cannot be disentangled. These findings highlight the necessity of assay- and population-specific validation to ensure precise clinical application.

To further contextualize our findings with previously published dd-cfDNA studies, we compared the diagnostic performance of our assay with several representative studies (Supplementary Material, Supplementary Section S5.3; Supplementary Table S3). Our assay demonstrated an AUC of 0.874 and a sensitivity of 81.0% at the 0.33% threshold, which is broadly comparable to the performance reported in the GRAfT study (AUC ∼0.85) and the DEDUCE study (AUC 0.86), both of which utilized SNP-based NGS platforms in predominantly Western populations [11, 23]. In contrast, Rodgers et al. reported lower sensitivity (39%) for their SNP-based dd-cfDNA assay in a mixed cohort, although the specificity was comparable (82%–84%) [24]. These variations in performance metrics are likely influenced by multiple factors, including assay design (Indel vs. SNP), cohort composition (for-cause vs. surveillance/mixed), prevalence of rejection, and differences in study populations, rather than population differences alone. Notably, our assay achieved a higher sensitivity than the SNP-based assay reported by Rodgers et al. [24], which may be attributable to the use of population-optimized Indel markers in the East Asian genetic context.

Beyond AUC and sensitivity, a crucial distinction lies in the negative predictive value (NPV), which is heavily influenced by the prevalence of rejection in the study cohort. In surveillance-oriented cohorts such as GRAfT, DEDUCE, FreeDNA-CAR, and D-OAR [10, 11, 23, 24], the low prevalence of rejection (typically <20%) yields NPVs of ≥90% and often above 94%, supporting the use of dd-cfDNA as a rule-out test in routine monitoring. In contrast, our for-cause biopsy cohort had a high prevalence of rejection (∼60%), which inflates the PPV but reduces the NPV to 75% (with a bootstrap lower bound of 53%). Therefore, in a high-pretest-probability setting such as for-cause biopsy, the 0.33% threshold is better suited for a rule-in strategy, whereas its utility as a rule-out test in routine surveillance would require validation in lower-prevalence cohorts. Collectively, these comparative analyses reinforce that dd-cfDNA performance characteristics, including both sensitivity/specificity and predictive values, are influenced by the intended use (surveillance vs. for-cause testing), assay methodology, and patient population. Direct head-to-head comparisons using identical cohorts and standardized protocols are warranted to further elucidate these differences, and caution is needed when extrapolating our findings to different clinical settings. A key finding was the strong association between dd-cfDNA levels and histological injury severity. The significant elevations in ACR ≥2R and AMR ≥2, but not in ACR 1R, reinforce dd-cfDNA’s role as a biomarker for clinically significant graft injury. The numerical trend toward higher dd-cfDNA levels in AMR compared to ACR is biologically plausible. AMR, primarily mediated by humoral immunity, often causes endothelial damage, which is a rich source of cfDNA. This pattern has been observed in other solid organ transplants and warrants investigation in larger, AMR-focused studies [10]. However, the small number of AMR events (n = 9) limits the reliability of these subgroup comparisons, and no definitive conclusions should be drawn regarding the differential performance of dd-cfDNA for ACR versus AMR based on this study alone. Longitudinal analysis revealed that dd-cfDNA levels approached a stable range from week 4, with stable levels reached by week 8. This timeframe is broadly consistent with the stabilization period described in the GRAfT study, which reported a 28-day (4-week) timeframe for dd-cfDNA to reach a stable baseline after transplantation [11]. Consequently, our data suggest that dd-cfDNA levels stabilize after 8 weeks post-transplant, and accordingly, caution should be exercised when interpreting dd-cfDNA-based signals obtained before this time point. However, given the relatively small cohort and limited follow-up, this observation should be confirmed in larger studies before any categorical recommendation regarding the optimal timing for clinical monitoring can be made.

Serial measurements of dd-cfDNA have provided preliminary pathophysiological insights into the responses to rejection therapy. In our cohort treated with methylprednisolone-based regimens, dd-cfDNA levels decreased significantly following anti-rejection therapy. This reduction was paralleled by improvements in NT-proBNP levels and CD4^+^ lymphocyte depletion. The parallel decline in dd-cfDNA alongside clinical improvement suggests that dd-cfDNA may serve as a real-time indicator of graft injury resolution during anti-rejection therapy. When stratified by rejection type, dd-cfDNA decreased significantly in the ACR subgroup at the two-week assessment, whereas the decrease in the AMR subgroup was more modest and did not reach statistical significance. This observation suggests that for AMR, the resolution of graft injury as reflected by dd-cfDNA decline may require a longer time course than the two-week window assessed in this study. However, given the small sample size and the absence of post-treatment biopsy confirmation, these exploratory findings should be interpreted with caution, and the optimal timing for dd-cfDNA assessment after AMR treatment warrants further investigation. Furthermore, the lack of significant change in LVEF despite improvements in dd-cfDNA levels may reflect a delayed structural response following injury resolution. Collectively, these findings support the potential utility of dd-cfDNA for monitoring treatment response, and suggest that the kinetics of dd-cfDNA recovery may differ between ACR and AMR, with AMR potentially requiring a longer observation window. These preliminary observations warrant validation in larger prospective studies.

Moreover, dd-cfDNA exhibited a graded inverse correlation with graft systolic function, with the highest levels observed in cases of severe dysfunction (LVEF <50%), intermediate levels in mild impairment (LVEF 50%–60%), and the lowest levels in preserved function (LVEF ≥60%). This relationship was further supported by a weak but significant inverse association in GEE models. Notably, a stratified analysis revealed that this inverse association persisted in the AR subgroup but was absent in the NR subgroup, suggesting that the overall correlation is largely driven by acute rejection. This finding supports the interpretation that dd-cfDNA specifically reflects immune-mediated graft injury rather than serving as a general marker of cardiac dysfunction. However, the limited number of observations in the severely reduced LVEF group warrants caution in interpreting the categorical findings.

Nevertheless, the relationship between dd-cfDNA and LVEF was not uniform across all patients, and several discordant patterns were observed that further inform the interpretation of dd-cfDNA levels in relation to graft injury. First, elevated dd-cfDNA levels were noted in patients with biopsy-confirmed moderate-to-severe ACR (≥2R) despite preserved LVEF, suggesting that dd-cfDNA elevation may precede functional deterioration. This observation aligns with recent evidence that dd-cfDNA elevation is associated with an increased risk of subsequent graft dysfunction, and that dd-cfDNA may be more sensitive than conventional echocardiography for detecting early graft injury [25–27]. Second, patients with reduced LVEF but without dd-cfDNA elevation were found to have no rejection on biopsy, suggesting that the LVEF reduction in these cases may be attributable to non-myocardial injury factors such as altered loading conditions or arrhythmias. Third, a small subset of patients exhibited elevated dd-cfDNA with preserved LVEF but only low-grade or no rejection, often with prominent Quilty lesions. While the significance of this finding remains uncertain, it has been suggested that certain Quilty phenotypes may reflect subclinical immune activity [28, 29]. It is also worth noting that endomyocardial biopsy, despite being the gold standard, has well-documented sampling error and interobserver variability [1, 30]. In this context, the discordant cases we observed may partly reflect these inherent limitations, raising the possibility that dd-cfDNA could detect subclinical graft injury missed by EMB. Collectively, these observations support dd-cfDNA as a direct marker of myocardial injury rather than a surrogate for cardiac function. This distinction enhances its clinical utility, particularly in detecting early or subclinical allograft injury before functional decline becomes apparent. However, these observations are based on case-level descriptions (Supplementary Material, Supplementary Section S5.4; Supplementary Table S4) and require confirmation in prospective studies.

To facilitate clinical implementation within the Chinese context, we have developed an innovative NGS-based assay utilizing a panel of 48 insertion-deletion (Indel) markers specifically optimized for East Asian genetic backgrounds. This selection of markers was informed by minor allele frequencies (MAF 0.4–0.6 in East Asians) and the characteristics of flanking sequences. Previous studies have predominantly employed SNP-based methodologies for dd-cfDNA detection [13, 31]. Compared to SNP-based approaches, our Indel-focused design may overcome limitations associated with single-base resolution of NGS and reduce background noise. Additionally, the multiplexing capability of NGS surpasses that of digital PCR (dPCR) by enabling simultaneous multi-locus assessment, which mitigates the risks associated with single-marker failure and enhances assay robustness [32, 33]. Paired-end sequencing further ensures the accurate discrimination of true Indel variants from sequencing artifacts.

Based on the preliminary validation of our locally developed assay and the established threshold in this exploratory study, we suggest that dd-cfDNA monitoring may be integrated as an adjunctive tool in heart transplant surveillance protocols for Asian populations, with monitoring initiated after the early postoperative phase to allow for resolution of graft injury associated with ischemia-reperfusion injury. A cutoff value of 0.33% may be employed to inform clinical decision-making. However, the clinical utility of this threshold must be interpreted in the context of the observed performance metrics. The high PPV (89.5%) is expected given the elevated prevalence of rejection (60%) in our for-cause biopsy cohort, and caution is warranted when extrapolating this value to routine surveillance settings where the prevalence of rejection is lower. Conversely, the NPV (75.0%) is substantially lower than the ≥94% reported in previous surveillance studies [11, 21, 23, 34, 35], with a lower confidence bound of 52.94% in bootstrap validation, indicating that dd-cfDNA at this threshold is insufficient to safely rule out clinically significant rejection. Therefore, dd-cfDNA is more suitable as a “rule-in” test than a “rule-out” test: elevated levels (≥0.33%) should trigger comprehensive evaluation, whereas low values should not be used in isolation to defer biopsy but interpreted alongside clinical assessment and other biomarkers. Collectively, these observations support dd-cfDNA as a direct marker of donor myocardial injury, detectable before systolic dysfunction becomes apparent, which may facilitate earlier identification of allograft injury and provide additional context for evaluating graft dysfunction. Future studies are needed to externally validate these findings and assess the clinical utility of dd-cfDNA-guided monitoring in broader populations.

This study has several limitations that should be considered when interpreting its findings. First, the single-center design and limited sample size, particularly the small number of AMR cases, may limit the generalizability of the results. Of note, infection was significantly more frequent in the rejection group than in the non-rejection group (30.0% vs. 0%, P = 0.031), and the limited sample size precluded adjustment for this and other potential confounders such as infection, which may elevate dd-cfDNA levels independently of rejection. This potential confounding should be considered when interpreting the observed associations. Second, since the reference cohort did not undergo protocol biopsies, the possibility of misclassifying subclinical rejection as “rejection-free” cannot be excluded, which may have influenced the estimated baseline levels and the derived threshold. Third, the biopsy cohort consisted exclusively of patients with clinical suspicion of rejection, which increases the pre-test probability and may affect the generalizability of the diagnostic performance estimates to routine surveillance settings. Fourth, the heterogeneity in post-transplant time at biopsy and the limited follow-up beyond 1 year restrict our ability to assess the stability of the threshold across different post-transplant periods. Additionally, the small sample size for treatment response analysis limits the robustness of these observations. Finally, the absence of direct comparison with commercial assays available in other regions prevents benchmarking against international platforms. Despite these limitations, this study represents one of the first comprehensive evaluations of dd-cfDNA in a Chinese heart transplant population, with a relatively large single-center cohort and a broad spectrum of analyses encompassing longitudinal kinetics, rejection phenotyping, treatment response, and graft function. Future multicenter studies with larger sample sizes, protocol biopsies, and extended follow-up are warranted to validate these findings and refine the clinical application of this assay.

## Conclusion

This exploratory study provides initial evidence that dd-cfDNA, measured using a domestically developed Indel-based assay, may serve as a noninvasive biomarker for detecting acute rejection in Chinese heart transplant recipients. An assay- and cohort-specific threshold of 0.33% was identified, with diagnostic performance broadly comparable to that reported in international studies. The association between dd-cfDNA levels and histological injury severity, along with its kinetic changes following treatment, supports its potential as an adjunctive tool in rejection monitoring. Given the modest NPV, the current threshold appears better suited for a “rule-in” rather than a “rule-out” strategy, and low values should not be used in isolation to defer biopsy. When used as part of a multimodal surveillance strategy, dd-cfDNA monitoring could potentially contribute to reducing reliance on invasive biopsies, facilitating earlier therapeutic intervention, and potentially improving long-term graft outcomes. However, these findings are exploratory and should not be interpreted as evidence that dd-cfDNA can safely replace or reduce the need for endomyocardial biopsy; prospective validation in larger, multicenter cohorts and routine surveillance settings is required before any clinical implementation.

## Data Availability

The original contributions presented in the study are included in the article/Supplementary Material, further inquiries can be directed to the corresponding author.
