# Supplementary material for: Prospective evaluation of donor-derived cell-free DNA for noninvasive detection of acute rejection in an Asian heart transplant population
Source: Transpl Int. 2026 Oct 5;39:16415. doi: 10.3389/ti.2026.16415 (PMC13677294; doi:10.3389/ti.2026.16415)
Supplement: Supplementary file 1 [file DataSheet1.pdf]

Supplemental Materials

S1. Criteria for Biopsy Indication

Table S1. Clinical Indications for For-Cause Endomyocardial Biopsy in the Biopsy Cohort[1]

Category	Clinicalindications
Cardiac dysfunction/ hemodynamic instability	LVEF decline, hypotension, elevated venous pressure, edema, dyspnea
ECG/ arrhythmic abnormalities	Frequent premature beats, high-grade AV block,R-wave progression loss, ventricular arrhythmias
Elevated biomarkers	Elevated NT-proBNP / BNP
Echocardiographic/ imaging abnormalities	Septal thickening, LV wall thickening, left atrial enlargement, pericardial effusion, myocardial edema on MRI
Immunologic abnormalities	DSA positivity, HLA class II positivity, PRA positivity
Clinical symptoms	Chest tightness, shortness of breath, reduced exercise tolerance, paroxysmal nocturnal dyspnea, cough, pulmonary congestion

S2. Detailed Treatment Protocol for Acute Rejection

Patients with biopsy-confirmed acute rejection (ACR ≥ 2R or AMR ≥ 1 per 2016 ISHLT criteria) received stratified treatment[2]. For isolated ACR ≥ 2R, primary treatment was intravenous pulse methylprednisolone (250-1000 mg/day for three days), followed by an oral prednisone taper and optimization of baseline immunosuppression (tacrolimus trough: 8-12 ng/mL; mycophenolate mofetil: 1.27±0.33 g/day). Refractory ACR received rabbit anti-thymocyte globulin (0.75-1.5

mg/kg/day for 3-5 days).

For AMR ≥ 1, personalized combination therapy was administered based on severity and DSA titers, including: 1) Plasmapheresis (3-5 sessions) or high-dose IVIG (2 g/kg); 2) Rituximab (375 mg/m² weekly for 1-4 doses); 3) Eculizumab (900 mg weekly for four doses in C5b-9 positive cases). Mixed rejection (ACR ≥ 2R + AMR ≥ 1) was treated with corticosteroids plus AMR-directed therapy. Response was evaluated via follow-up EMB 7-14 days post-therapy and DSA screening.

S3. Detailed Laboratory Methods for dd-cfDNA Quantification

Blood Collection and cfDNA Extraction

A sufficient blood volume (8 mL) was collected into cell-free DNA BCTs (Streck). Plasma separation was performed by centrifugation at 1600 × g for 10 minutes at 4°C. The supernatant underwent secondary centrifugation at 16,000 × g for 10 minutes at 4°C. CfDNA was immediately extracted from 1.8 mL of supernatant using the QIAamp Circulating Nucleic Acid Kit (Qiagen). Separated plasma was stored at −80°C for batch analysis.

Indel Marker Selection and Primer Design

Polymorphic Indels for the East Asian population were screened from the 1000 Genomes Project (GRCh38). Sites with two alleles and minor allele frequency (MAF 0.4-0.6) were selected. Bioinformatic filtering enforced a minimum 3 Mb inter-locus distance, excluded repetitive regions and immune gene clusters, ensured SNP-free flanking regions (MAF<0.1), and required 45-55% GC content. This yielded 48-plex Indel markers. PCR primers were designed for 70-90 bp amplicons to ensure read

overlap in PE100 sequencing.

Targeted Amplification and Library Sequencing

cfDNA input (>20 ng) was used for a single multiplex PCR (15 cycles) with the 48-plex primer panel. A second PCR incorporated Illumina adapters and indices. Sequencing required a minimum depth of >10,000x per locus. Samples failing QC were repeated.

dd-cfDNA Quantification and Bioinformatics

Raw sequencing data were trimmed and aligned to GRCh38 using BWA. Indels were called using Samtools. For each informative locus, allele counts were used to calculate the minor allele ratio. Final dd-cfDNA percentage was derived after PCR bias and background correction. The assay is precise within 0.05% - 1.5% (Figure S1). Samples with failed quality control analysis were repeated for library construction and sequenced, and all dd-cfDNA quantification results were output as final results after PCR bias correction and background value improvement.

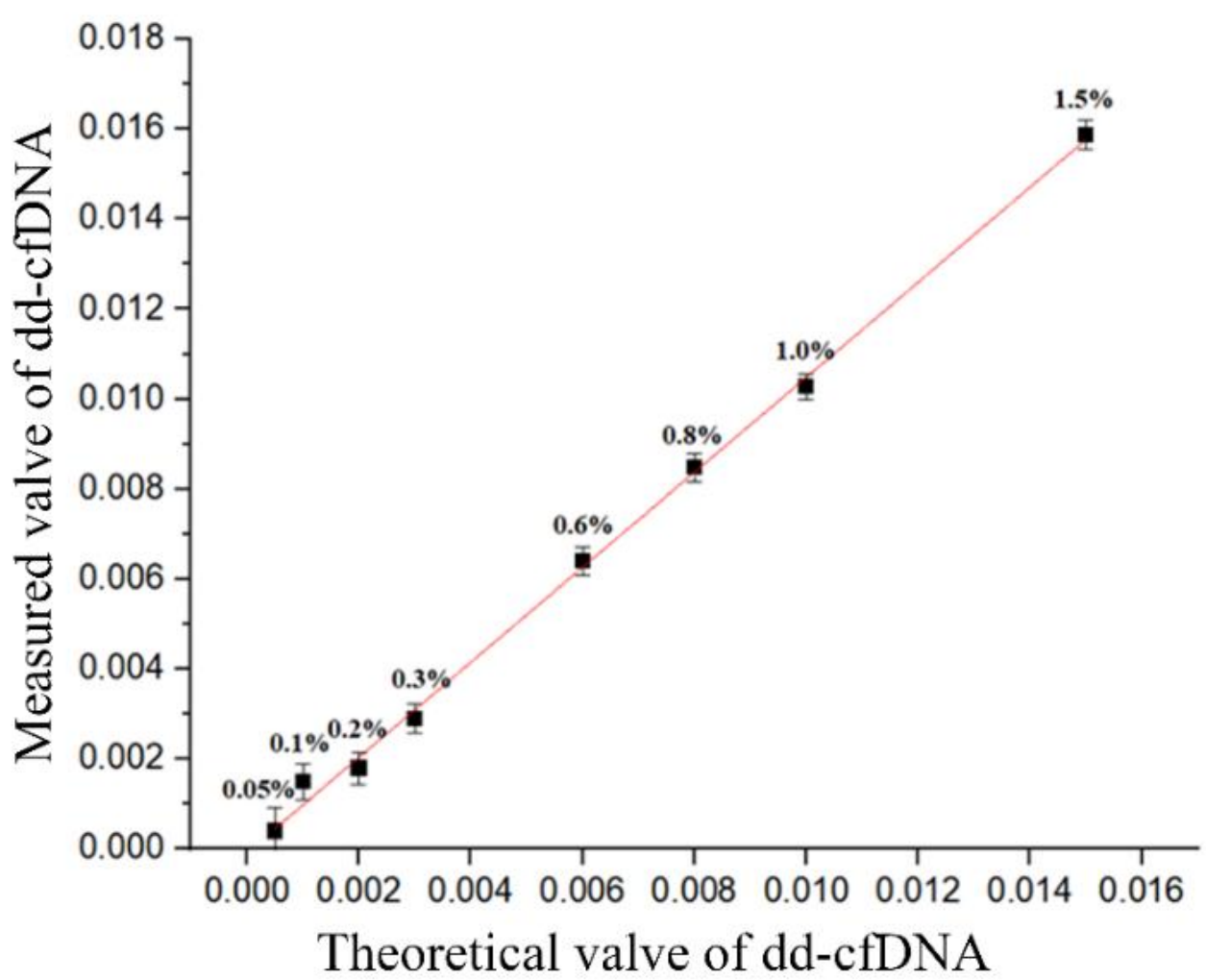

Figure S1. Shows the linearity and accuracy of dd-cfDNA assay in reference

standards samples and blank samples. Indels targetion sequencing was used to predicte dd-cfDNA percentage (y-axis). The X axis represents the theoretical concentration of dd-cfDNA. A linear fit to these points has a slope of 0.83, and R^2 value of 0.988.

S4. Detailed Statistical Analysis Methods

The primary objective was to evaluate donor-derived cell-free DNA (dd-cfDNA) ability to discriminate acute rejection (AR) from no rejection (NR), with analyses performed separately for the reference cohort and the biopsy-paired cohort according to their study designs.

Reference cohort (longitudinal repeated measures)

Each patient contributed multiple dd-cfDNA samples at predefined post- transplant time points (weeks 1, 2, 4, 8, 24, and 48). Temporal dynamics were analyzed using generalized estimating equations (GEE) with an exchangeable correlation structure and patient ID as the clustering variable. Pairwise comparisons between time points were performed using estimated marginal means with Holm-Bonferroni adjustment to account for within-subject correlation and missing data.

Biopsy cohort: cross-sectional group comparisons (independent observations)

For group comparisons (e.g., AR vs. NR; acute cellular rejection [ACR] grade 0R vs. 1R vs. ≥2R; antibody-mediated rejection [AMR] grade 0 vs. 1 vs. ≥2), each patient contributed only one sample (the first biopsy during the study period, or the biopsy corresponding to the most severe rejection grade if multiple biopsies occurred). Thus, observations were independent. Continuous variables were compared using the

Kruskal-Wallis test for three or more groups, followed by post-hoc pairwise comparisons using the Dwass-Steel-Critchlow-Fligner (DSCF) test. For two-group comparisons, the Mann-Whitney U test was used. Categorical variables were compared using Fisher‘s exact test. Effect sizes for significant differences were quantified using Hodges-Lehmann estimators (median differences) with 95% confidence intervals.

Biopsy cohort: Pre- vs. Post- therapy comparisons (paired repeated measures)

For patients who received anti-rejection therapy and had dd-cfDNA measured both before and after treatment (paired samples), the Wilcoxon signed-rank test was used. These analyses were performed separately for the ACR and AMR subgroups and are reported as exploratory given the small sample sizes.

Association analysis

The association between dd-cfDNA levels and left ventricular ejection fraction (LVEF) was assessed using GEE models with an exchangeable correlation structure, with LVEF treated as both a categorical variable (three groups: <50%, 50–60%, ≥60%) and a continuous variable. To address potential confounding by acute rejection and repeated echocardiographic assessments, a subgroup analysis stratified by rejection status (AR vs. NR) was performed using GEE as the primary confirmatory approach. This approach accounts for within-subject correlation across repeated measurements and provides robust estimates of the association between LVEF and dd-cfDNA levels.

Diagnostic performance (biopsy cohort, cross-sectional)

Because each patient contributed only one dd-cfDNA measurement for the diagnostic analysis (paired with the corresponding biopsy), standard receiver operating characteristic (ROC) curve analysis was performed. The optimal threshold was determined using Youden's index. To internally validate the performance metrics, hierarchical (block) bootstrapping with 1,000 resamples was conducted at the patient level (i.e., resampling patients with replacement while retaining all samples from a resampled patient) to account for the fact that a small number of patients contributed multiple biopsies over time. Bias‐ corrected 95% confidence intervals for sensitivity, specificity, positive predictive value (PPV), and negative predictive value (NPV) were derived from the bootstrap distribution.

Sample size considerations

No a priori sample size calculation was performed; the sample size was determined by the number of eligible patients during the enrollment period. The main diagnostic analysis (AUC = 0.874) was adequately powered with 21 AR events, but subgroup analyses (particularly for AMR, n = 9) are exploratory due to limited statistical power, as acknowledged in the Limitations.

All statistical tests were two-sided, with $P < 0.05$ considered statistically significant. Analyses were performed using R version 4.2.2 (R Foundation for Statistical Computing, Vienna, Austria). No custom code is provided; all analyses used standard R functions and packages (e.g., “geepack” for GEE, “rms” for mixed effects, “pROC” for ROC analysis, “boot” for hierarchical bootstrapping).

S5. Supplementary Results

S5.1 Detailed Patient and Sample Flow

Study Population Overview

A total of 80 heart transplant recipients were enrolled in this prospective study. Patients were assigned to two analytical cohorts based on the type of dd-cfDNA samples collected:

1. Reference Cohort (n = 42)

- Comprised clinically stable patients without signs or symptoms of rejection.
- Contributed 243 longitudinal dd-cfDNA samples collected at scheduled post-transplant timepoints: weeks 1, 2, 4, 8, 24, and 48.
- Protocol (surveillance) biopsies were not performed in this cohort per institutional practice; absence of rejection was assumed based on clinical stability.

2. Biopsy Cohort (n = 41)

- Comprised patients undergoing for-cause endomyocardial biopsy due to clinical suspicion of rejection.
- A total of 42 biopsy events were performed in 41 patients. One patient underwent two biopsies (at 2 years and 3 years post-transplant), both confirming AMR grade 2.
- These 42 biopsy events yielded 42 pre-biopsy dd-cfDNA samples. An additional 14 post-therapy samples were collected from 14 patients following anti-rejection treatment, bringing the total number of biopsy-paired samples to 56.
- Based on biopsy findings, the 42 biopsy events were categorized into four groups: NR (ACR 0R, AMR 0; n = 14), ACR 1R (n = 7), ACR ≥2R (n = 11), and AMR (AMR ≥1, including mixed rejection; n = 10 from 9 patients, with one patient contributing two biopsies at different timepoints).

3. Overlap Between Cohorts

Three patients from the reference cohort developed clinical suspicion of rejection during the one-year follow-up period and underwent for-cause biopsy, thereby entering the biopsy cohort.

Importantly, the dd-cfDNA samples collected from these patients for the reference cohort were obtained at scheduled surveillance timepoints (weeks 1, 2, 4, 8, 24, and 48) and were entirely independent from the pre-biopsy samples collected at the time of for-cause biopsy. These independent samples served distinct analytical purposes: longitudinal samples for kinetics analysis and pre-biopsy samples for diagnostic performance evaluation.

Therefore, these 3 patients were counted in both cohorts, contributing to the total of 80 unique patients overall (42 + 41 − 3 = 80).

S5.2 Table S2. Estimated marginal means of dd-cfDNA levels from generalized estimating equations (GEE)

Time point	No. of samples	Estimated marginal mean (%)	95% CI (%)	P value (vs. Week 8)*
Week 1	42	0.372	0.271–0.473	0.042
Week 2	42	0.301	0.248–0.354	0.042
Week 4	42	0.309	0.221–0.397	0.330
Week 8	40	0.207	0.171–0.242	Reference
Week 24	39	0.222	0.187–0.257	1.000
Week 48	38	0.371	0.208–0.534	0.569

*P values adjusted for multiple comparisons using Holm-Bonferroni method.
Footnotes: GEE with exchangeable correlation structure, accounting for within-subject correlation and missing data.

S5.3 Table S3.Comparison of diagnostic performance of dd-cfDNA for acute rejection detection across major studies

Study	Population	N (patients/ samples)	Assay platform	Threshold for AR	Sensitivity	Specificity	AUC	Study significance/ Key conclusion
Present study	Chinese	80 / 299	Indel-based NGS (48 markers)	0.33%	81.00%	85.70%	0.874	• First validation of Indel-based dd-cfDNA assay in Chinese HTx cohort; establishes assay- and cohort-specific threshold (0.33%)
Agbor-Enoh et al.[3] 2021 (GRAfT)	Mixed (US, multicenter)	171 / 1,834	SNP-based NGS	0.20% – 0.25%	~65%	~85%	~0.85	• %dd-cfDNA detects AR with high AUC and NPV; supports use of dd-cfDNA to monitor for AR and paves way for clinical utility studies
Kim et al. 2022 [4] (DEDUCE)	Mixed (US)	223 / 811	SNP-based NGS (Prospera™)	0.15%	78.50%	76.90%	0.86	• Novel dd-cfDNA test detects AR with good accuracy; holds promise as noninvasive test for AR in HTx recipients
Kim et al. 2025 [5] (2-Threshold)	Mixed (US)	187 / 808	SNP-based NGS (Prospera™ with DQS)	2TA*: 0.26% or DQS⩾18	86.50%	83.60%	0.881	• Two-threshold algorithm (dd-cfDNA fraction + DQS) improves detection of clinically significant rejection
Jiménez-Blanco et al. 2025 [6]	Mixed (Spain, 12	206 / 1,090	SNP-based NGS (Allonext)	0.10%	N/A	N/A	0.603	• dd-cfDNA may be a useful biomarker to rule out significant ACR in low-risk population;

Study	Population	N (patients/ samples)	Assay platform	Threshold for AR	Sensitivity	Specificity	AUC	Study significance/ Key conclusion
(FreeDNA-CAR)	centers)							combination with NT-proBNP could reduce >50% of EMBs
Böhmer et al. 2025[7]	Mixed (adults + pediatrics)	94 / 1,007	ddPCR (absolute quantification)	25 copies/mL**	N/A	N/A	0.87***	• Absolute quantification with ddPCR is fast and effective for monitoring graft health; helps identify non-rejection factors influencing cfDNA levels
Liu et al. 2025[8] (Meta-analysis)	Pooled (10 studies)	N/A	Multiple platforms	Pooled across studies	65%	79%	0.83	• Plasma dd-cfDNA may serve as a promising non-invasive biomarker for AR diagnosis in HTx recipients

*2TA: two- threshold algorithm combining dd- cfDNA fraction and Donor Quantity Score (DQS).
**Threshold for detecting symptomatic rejection; for overall rejection, AUC was 0.68.
***AUC for symptomatic rejection (AUC 0.87); AUC for overall rejection was 0.68.
N/A = not available or not reported.

Footnotes:
AR = acute rejection; HTx = heart transplantation; NGS = next- generation sequencing; SNP = single nucleotide polymorphism; Indel = insertion/deletion; ddPCR = droplet digital PCR; DQS = Donor Quantity Score; 2TA = two- threshold algorithm; AUC = area under the receiver operating characteristic curve; NPV = negative predictive value; EMB = endomyocardial biopsy.

S5.4 Table S4. Cases with diagnostically significant discordance between dd-cfDNA levels and graft systolic function (LVEF).

Case Pattern	Case number	dd-cfDNA	LVEF	Biopsy Timepoint Post-Transplant	Biopsy Findings	Biopsy Indication
**Pattern 1: Elevated dd-cfDNA with Preserved LVEF**	No. 1	0.91%	68%	1 year ~5 year	ACR 0R, AMR 0, Quilty	Bilateral lower extremity edema with fatigue and with elevated NT-proBNP
	No. 2	0.35%	62%	>5 year	ACR 2R, AMR 0	Elevated NT-proBNP
	No. 3	0.81%	60%	>5 year	ACR 2R, AMR 0	Echocardiographic evidence of left atrial enlargement
	No. 4	1.04%	66%	>5 year	ACR 0, AMR 2	Echocardiographic evidence of left atrial enlargement, interventricular septal and left ventricular wall thickening, with a small amount of pericardial effusion
	No. 5	1.99%	63%	1 year ~5 year	ACR 1R, AMR 0, Quilty	HLA antibody positive
	No. 6	0.69%	60%	< 1 year	ACR 1R, AMR 2	Intermittent high-grade atrioventricular block with escape rhythm on Holter monitoring
	No.7	0.38%	71%	< 1 year	ACR 0, AMR 0	Echocardiographic evidence of interventricular septal thickening
	No. 8	0.73%	65%	1 year ~5 year	ACR 1R, AMR0，Quilty	Dyspnea
	No. 9	0.94%	60%	< 1 year	ACR 2R, AMR 0, with perioperative	Echocardiographic evidence of interventricular septal thickening and with

					Ischemia-Reperfusion Injury	elevated NT-proBNP
	No. 10	1.48%	63%	< 1 year	ACR 0R, AMR 2	Intermittent palpitations, echocardiography showing significant ventricular wall thickening and left atrial enlargement, with elevated NT-proBNP
	No. 11	2.48%	60%	< 1 year	ACR 1R, AMR 2	Elevated venous pressure with hypotension
	No. 12	1.31%	61%	< 1 year	ACR 3R, AMR 0	HLA class II antibodies detected
**Pattern 2: Reduced LVEF with Low dd-cfDNA**	No. 13	0.27%	33%	>5 year	ACR 1R,AMR 0	Reduced exercise tolerance following an upper respiratory infection
	No. 14	0.16%	47%	>5 year	ACR0, AMR 0，Quilty	Chest tightness, shortness of breath, paroxysmal nocturnal dyspnea, and bilateral lower extremity edema
	No. 15	0.12%	27%	< 1 year	ACR0, AMR 0	Echocardiography revealed significant impairment of cardiac function

References:

1. Velleca A, Shullo MA, Dhital K, et al. The International Society for Heart and Lung Transplantation (ISHLT) guidelines for the care of heart transplant recipients. *J HEART LUNG TRANSPL*. 2023; **42**: e1-141.
2. Mehra MR, Canter CE, Hannan MM, et al. The 2016 International Society for Heart Lung Transplantation listing criteria for heart transplantation: A 10-year update. *J HEART LUNG TRANSPL*. 2016; **35**: 1-23.
3. Agbor-Enoh S, Shah P, Tunc I, et al. Cell-Free DNA to Detect Heart Allograft Acute Rejection. *CIRCULATION*. 2021; **143**: 1184-97.
4. Kim PJ, Olymbios M, Siu A, et al. A novel donor-derived cell-free DNA assay for the detection of acute rejection in heart transplantation. *J HEART LUNG TRANSPL*. 2022; **41**: 919-27.
5. Kim PJ, Olympios M, Sideris K, et al. A two-threshold algorithm using donor-derived cell-free DNA fraction and quantity to detect acute rejection after heart transplantation. *AM J TRANSPLANT*. 2025; **25**: 1895-905.
6. Jiménez-Blanco M, Crespo-Leiro MG, García-Cosío Carmena MD, et al. Donor-derived cell-free DNA as a new biomarker for cardiac allograft rejection: A prospective study (FreeDNA-CAR). *The Journal of heart and lung transplantation : the official publication of the International Society for Heart Transplantation*. 2025; **44**: 560-9.
7. B Hmer J, W Hlander HK, Tran-Lundmark K, et al. Absolute quantification of donor-derived cell-free DNA following pediatric and adult heart transplantation. *The Journal of heart and lung transplantation : the official publication of the International Society for Heart Transplantation*. 2025; **44**: 1638-47.
8. Liu S, Wang L, Liu S, Zhao Y. Donor-derived cell-free dna as a diagnostic biomarker for acute rejection in heart transplantation: A systematic review and meta-analysis. *Transplantation reviews (Orlando, Fla.)*. 2025; **39**: 100916.
